# Supplementary material for: Manual Chest Compression versus Automated Chest Compression Device during Day-Time and Night-Time Resuscitation Following Out-of-Hospital Cardiac Arrest: A Retrospective Historical Control Study
Source: J Pers Med. 2023 Jul 28;13(8):1202. doi: 10.3390/jpm13081202 (PMC10455266; doi:10.3390/jpm13081202)
Supplement: Supplementary file 1 [file jpm-13-01202-s001.zip › jpm-2502938-supplementary.pdf]

Supplementary Table S1: Interaction between in-hospital ACCD use and admission time on outcomes

Outcomes	p for interaction
ROSC	< 0.001
Chest injuries	< 0.001
IHCPR duration	< 0.001
Survival to ED discharge	< 0.001
Survival to hospital discharge	< 0.001
Survival with good neurological outcome to hospital discharge	< 0.001

ACCD, automatic chest compression devices; ROSC, return of spontaneous circulation; IHCPR, in-hospital cardiopulmonary resuscitation; ED, emergency department.
